# Retinal Microcirculatory Responses to Hyperoxia in Primary Open-Angle Glaucoma Using Optical Coherence Tomography Angiography

**DOI:** 10.1167/iovs.62.14.4

**Published:** 2021-11-03

**Authors:** Xintong Fan, Huan Xu, Ruyi Zhai, Qilian Sheng, Xiangmei Kong

**Affiliations:** 1 Eye Institute and Department of Ophthalmology, Eye & ENT Hospital, Fudan University, Shanghai, China; 2 NHC Key Laboratory of Myopia (Fudan University); Key Laboratory of Myopia, Chinese Academy of Medical Sciences, Shanghai, China; 3 Shanghai Key Laboratory of Visual Impairment and Restoration, Shanghai, China

**Keywords:** optical coherence tomography angiography, open-angle glaucoma, retinal vascular response, radial peripapillary capillary, hyperoxia

## Abstract

**Purpose:**

To investigate the retinal vascular response to hyperoxia in patients with primary open-angle glaucoma (POAG) using optical coherence tomography angiography (OCTA).

**Methods:**

This prospective study included 27 eyes in 27 patients with POAG and 14 eyes in 14 age- and sex-matched healthy participants. Retinal radial peripapillary capillary (RPC) perfusion was measured by OCTA before and after inhaling oxygen in all participants. Systemic hemodynamic variables were also examined and recorded before and after hyperoxia.

**Results:**

Hyperoxia significantly reduced the perfused vessel density (PVD) of RPCs in both healthy controls (baseline and hyperoxia: 54.2 ± 4.1 and 51.0 ± 4.4, respectively, *P* < 0.001) and patients with POAG (baseline and hyperoxia: 44.7 ± 6.1 and 43.2 ± 5.4, respectively, *P* = 0.001). However, the changes in peripapillary PVD between the two gas conditions in patients with POAG were significantly lower than in healthy controls, including both the absolute change (baseline-hyperoxia: 1.5 ± 2.0 and 3.2 ± 1.2, respectively, *P* = 0.006) and relative change (ratio of absolute change and baseline value: 3.0% ± 4.6% and 6.0% ± 2.4%, respectively, *P* = 0.04).

**Conclusions:**

Retinal microvasculature responds to hyperoxia by reducing RPC perfusion in both healthy participants and patients with POAG. However, this vasoreactivity capacity was significantly impaired in patients with POAG.

Primary open-angle glaucoma (POAG), a leading cause of irreversible blindness worldwide,^1^ is a neurodegenerative disease characterized by retinal ganglion cell damage and related visual field loss.^2^^,^^3^ Abnormally high intraocular pressure (IOP) has been recognized as a primary risk factor for POAG, which does not account for the presence of normal-tension glaucoma and the progression of optic nerve damage in patients with normal IOP.^4^ Another major factor contributing to the development of glaucoma is abnormal ocular blood flow.^5^ The reduction of ocular blood flow has been shown to be highly associated with POAG progression.^6^^,^^7^

Previous studies have observed impaired vascular reactivity in cerebral arteries,^8^ retrobulbar vessels,^9^^,^^10^ and retinal vessels^11^^–^^14^ in patients with POAG. However, most studies focused on large arteries or veins, while few have focused on the autoregulation of the retinal capillary system. Moreover, many vascular-related oculopathies develop primarily in microvessels. Previous studies have applied laser Doppler flowmetry to assess the microcirculation of the optic disc^10^^,^^12^; however, this technique is slow and has limited reproducibility.^14^^,^^15^

Optical coherence tomography angiography (OCTA) is a noninvasive technology that provides high-contrast imaging of retinal microvasculature with excellent resolution.^16^ Radial peripapillary capillaries (RPCs) compose a capillary bed network in the retinal nerve fiber layer (RNFL), which indispensably supplies retinal ganglion cells (RGCs). Several OCTA studies have reported diminished perfused vessel density of RPCs in peripapillary areas of patients with POAG.^6^^,^^7^^,^^17^ However, alterations in the retinal vascular response as assessed by OCTA have not yet been investigated. We previously successfully used OCTA and oxygen inhalation to test the retinal microvascular reactivity in healthy adults, revealing a significant reduction of vessel density in peripapillary regions after hyperoxia, and the repeatability of this protocol was also confirmed.^18^

In this study, we extend this safe, noninvasive, and reproducible protocol to patients with POAG to investigate retinal microcirculatory responses in the optic nerve head (ONH) area in cases involving POAG and thus advance the understanding of the pathogenesis and progression of glaucoma.

## Methods

### Ethics Statement

This prospective study was approved by the Institutional Review Board of the Eye and Ear Nose and Throat (ENT) Hospital of Fudan University, Shanghai, China (No. 2014043), per the principles of the Declaration of Helsinki. Informed consent was obtained from all participants included in this study after an explanation of the nature and possible consequences of the study.

### Participants

Twenty-seven eyes of 27 patients with POAG who visited the Eye and ENT Hospital of Fudan University between August 2020 and October 2020 were recruited in this study. Fourteen eyes of 14 age- and sex-matched healthy participants with normal medical checkup reports were recruited as the control group from the community.

The inclusion criteria for healthy participants were as follows: (1) best-corrected visual acuity (BCVA) of 20/20 or better, (2) IOP <21 mm Hg, (3) a refractive error between −8.00 diopters (D) and +3.00 D, (4) normal findings on slit-lamp and fundus examinations, and (5) no history of IOP elevation, ocular diseases, intraocular surgery, or trauma. The inclusion criteria for patients with POAG were as follows: (1) a reliable visual field (VF) test with the presence of reproducible typical glaucomatous VF defects; (2) glaucomatous optic neuropathy with consistent VF defects, including optic disc changes and reduced RNFL thickness; (3) open angles examined by gonioscopy in both eyes; and (4) the existence of central fixation. The diagnosis of POAG was performed by a glaucoma specialist (XK). Participants with a history of other eye diseases such as retinopathy, neuro-ophthalmic diseases, or systemic diseases that may cause optic nerve damage were excluded from the study. One eye was selected randomly in each participant when both eyes met the inclusion criteria. All patients with POAG were treated with antiglaucoma agents during the disease course, and the IOP was within the normal range (<21 mm Hg) on the test day.

The exclusion criteria for both groups were as follows: (1) respiratory diseases or cardiopathy, which may prove hazardous during or after oxygen inhalation^19^^,^^20^; (2) systemic diseases that may affect ocular circulation such as hypertension, diabetes, or other microvascular disorders; and (3) significant cataracts (Lens Opacity Classification System III stage 2 or higher) or evident vitreous opacity.

### Ophthalmic Examinations

All participants underwent thorough ophthalmologic examinations, including BCVA, IOP (Goldmann applanation tonometry), refractive error, slit-lamp microscopy, gonioscopy, and fundus examination. RNFL and ganglion cell complex (GCC) thickness were measured using a spectral-domain OCT system (RTuve XR Avanti; Optovue, Fremont, CA, USA). VF testing was performed with a Humphrey field analyzer (Carl Zeiss Meditec, Dublin, CA, USA). Only high-quality OCT images (signal strength index >60) and VF tests with ≤33% fixation losses and false-negative and false-positive results were considered reliable and included in the study.

### Study Protocol

All participants refrained from caffeine and alcohol for at least 12 hours prior to examination.^21^ The following protocol has been described in detail in our previous study.^18^ Briefly, all participants were instructed to sit and breathe room air for 20 minutes before the oxygen inhalation test. Baseline characteristics, including blood pressure (BP), pulse rate, and retinal perfused vessel density in the ONH, were then measured and recorded. The oxygen flow rate was 15 L/min during the hyperoxia phase, which achieved an oxygen concentration of 80%.^22^^,^^23^ High-concentration oxygen masks (Intersurgical EcoLite; Intersurgical, Berkshire, UK) were applied for the oxygen inhalation. After 80% oxygen administration for 5 minutes, the BP, pulse rate, and retinal perfused vessel density of all participants were reevaluated, and the oxygen inhalation was maintained until the assessments were completed. The following formulas were used to calculate mean arterial pressure (MAP) and mean ocular perfusion pressure (MOPP): MAP = diastolic BP + 1/3 (systolic BP – diastolic BP); MOPP = 2/3 MAP – IOP. The reproducibility of our study protocol and the vessel density measurement have been checked and confirmed in our previous study.^18^

### OCTA Image Acquisition and Processing

All OCTA scans were obtained via a commercial OCTA device (RTuve XR Avanti; Optovue) with the RTVue-XR Avanti spectral-domain system using the split-spectrum amplitude-decorrelation angiography (SSADA) algorithm.^24^ The device used an A-scan rate of 70 kHz and provided an improved signal-to-noise ratio of blood flow detection. The software system automatically created an en face retinal angiogram through the internal projection of the flow signal to the retinal pigment epithelium. The B-scans (each consisting of 216 A-scans) were merged to create three-dimensional OCTA scans, and the motion artifacts were removed after the scan's acquisition. The scanning, which consisted of an optic disc region of 4.5 × 4.5 mm^2^, was repeatedly performed at the baseline and during the hyperoxia test. The segmentation of the retinal layer was automatically evaluated, and the RPC plexus from the inner limiting membrane to the RNFL was analyzed. The whole image refers to the entire 4.5 × 4.5-mm^2^ scanning area centered at the optic disc. The 2-mm-diameter inner circle consisting of the optic nerve head was defined as the inside disc, and the peripapillary region was defined as a 1-mm-wide annulus extending outward from the optic disc boundary (Fig.A).

**Figure.**
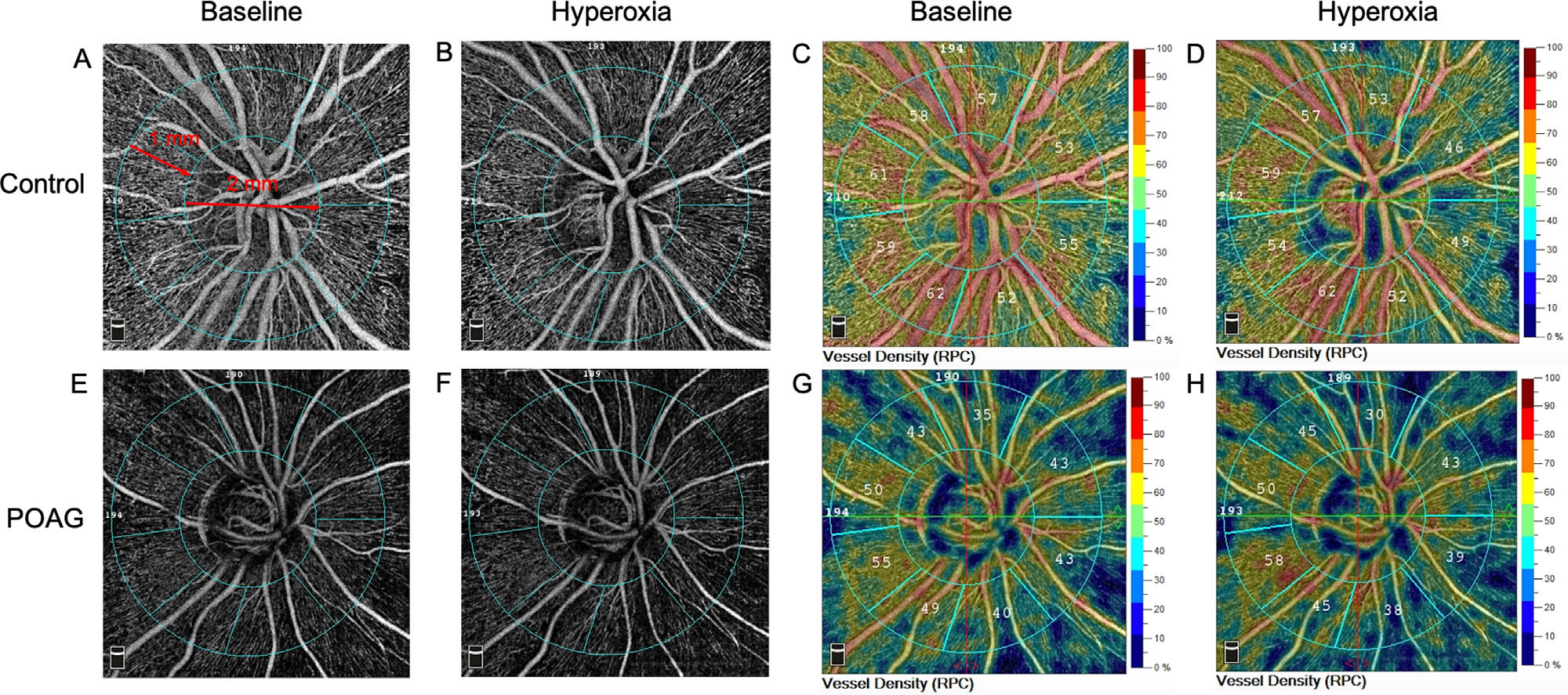
Examples of OCTA images and maps of RPC vessel density in eyes of a healthy control (**A–D**) and a patient with POAG (**E–H**) under baseline (**A**, **C**, **E**, **G**) and hyperoxia condition (**B**, **D**, **F**, **H**). The definition of the inside disc (2-mm-diameter inner circle) and peripapillary region (1-mm-wide annulus) in a 4.5 × 4.5-mm^2^ optic disc scan (whole image) is shown in panel **A**.

The large vessels were masked using the software so that only the capillary density was calculated, and the vessel density of different regions was automatically quantified using the custom software. The eye-tracking function was used, which improved the quality of the scanning image and largely reduced the residual motion artifacts. Only images with a signal strength index >8/10 and without motion artifacts were included in the analysis.

### Statistical Analysis

Data are expressed as mean ± standard deviation. The necessary sample size was determined by a power analysis, with the values of α = 0.05 and power = 0.80, and the proportion of POAG to healthy controls was 2:1. The calculated minimum sample size was 14 in the POAG group and 7 in the control group (MedSci Sample Size tools, MSST, version 6.0.2; MedSci, Shanghai, China). Student's *t*-tests and Mann–Whitney *U* (Wilcoxon rank-sum) tests were used to assess the differences between the control and POAG groups concerning continuous variables for normally distributed and nonnormally distributed data, respectively. Fisher's exact test was used to compare the proportions. Student's paired *t*-tests and Wilcoxon signed rank-sum tests were used to assess the differences between the baseline and hyperoxia values of systemic blood flow variables (normally distributed) and retinal perfused vessel density (nonnormally distributed), respectively. Univariate linear regression was used to analyze the association between BP and retinal perfused vessel densities. We used STATA version 15.1 (StataCorp, College Station, TX, USA) to perform the statistical analysis, and between-group differences were considered significant at *P* < 0.05.

## Results

### Baseline Information

Demographic and baseline clinical characteristics are shown in Table 1. A total of 27 patients with POAG (27 eyes) and 14 healthy controls (14 eyes) were included in this study. Both groups were comparable in age, gender, and blood flow variables, including systolic arterial pressure (SAP), diastolic arterial pressure (DAP), MAP, and MOPP. Surprisingly, the baseline pulse rate (PR) of patients with POAG was higher than that of healthy participants (*P* = 0.048). Univariate linear regression was conducted, and the results showed that PR was not associated with the outcomes of retinal perfused vessel density and retinal vascular responses (Supplementary Table S1). Therefore, the difference in baseline PR between the two groups did not affect our investigation regarding the effects of POAG on retinal microvascular responses to hyperoxia. Compared to healthy controls, patients with POAG exhibited a higher IOP (*P* = 0.03), worse BCVA (*P* = 0.002), larger cup-to-disc ratio (*P* < 0.001), thinner RNFL and GCC, smaller rim area, and larger focal loss volume percentage and global loss volume percentage (all *P* < 0.001). Reduced RPC vessel density was observed in patients with POAG in the whole image (*P* < 0.001), inside disc (*P* = 0.04), and the peripapillary region (*P* < 0.001).

**Table 1.** Demographic and Clinical Data at Baseline

Characteristic	POAG, n = 27	Control, n = 14	*P* Value
Age, y	41.2 ± 11.2	42.8 ±12.1	0.67
Male/female, *n*	12/15	6/8	1.00
Right/left, *n*	16/11	8/6	1.00
SAP, mm Hg	122.6 ± 16.9	121.5 ± 18.5	0.85
DAP, mm Hg	80.8 ± 10.6	78.9 ± 14.0	0.64
MAP, mm Hg	94.7 ± 12.1	93.1 ± 15.3	0.72
MOPP, mm Hg	47.1 ± 8.7	48.0 ± 9.9	0.75
PR, bpm	78.4 ± 12.4	70.6 ± 10.3	0.048
Disease duration, mo	29.6 ± 31.3	—	—
IOP, mm Hg	16.1 ± 3.3	14.1 ± 2.6	0.03
BCVA, logMAR	0.1 ± 0.2	−0.01 ± 0.03	0.002
Cup-to-disc ratio	0.6 ± 0.1	0.3 ± 0.2	<0.001
RNFL thickness, µm	79.2 ± 11.3	108.4 ± 18.9	<0.001
Rim area, mm^2^	0.8 ± 0.2	1.6 ± 0.6	<0.001
GCC thickness, µm	80.6 ± 11.4	100.8 ± 7.7	<0.001
FLV%	6.8 ± 4.7	0.5 ± 0.3	<0.001
GLV%	16.5 ± 10.4	1.9 ± 2.1	<0.001
Perfused vessel density			
Whole image	41.7 ± 4.9	50.8 ± 3.0	<0.001
Inside disc	44.8 ± 7.8	49.6 ± 4.9	0.04
Peripapillary	44.7 ± 6.1	54.2 ± 4.1	<0.001

Data are expressed as mean ± standard deviation unless otherwise indicated. FLV%, focal loss volume percentage; GLV%, global loss volume percentage; —, not applicable.

### Systemic Responses to Hyperoxia

Changes in blood flow variables after systemic hyperoxia are presented in Table 2. Blood pressure (including SAP, DAP, and MAP) and MOPP were not significantly altered after oxygen inhalation compared with baseline conditions in both the POAG and healthy control groups (all *P* > 0.05). However, the PR was significantly reduced after the hyperoxia test in both groups (all *P* < 0.001).

**Table 2.** Blood Flow Variables at Baseline and after Hyperoxia

	POAG, n = 27		Control, n = 14	
Characteristic	Mean ± SD	*P* Value	Mean ± SD	*P* Value
SAP, mm Hg				
Baseline	122.6 ± 16.9	—	121.5 ± 18.5	—
Hyperoxia	124.0 ± 14.6	0.38	123.9 ± 17.5	0.28
DAP, mm Hg				
Baseline	80.8 ± 10.6	—	78.9 ± 14.0	—
Hyperoxia	82.7 ± 10.9	0.13	81.4 ± 12.7	0.12
MAP, mm Hg				
Baseline	94.7 ± 12.1	—	93.1 ± 15.3	—
Hyperoxia	96.5 ± 11.6	0.15	95.6 ± 14.1	0.10
MOPP, mm Hg				
Baseline	47.1 ± 8.7	—	48.0 ± 9.9	—
Hyperoxia	48.2 ± 8.1	0.15	49.6 ± 9.4	0.10
PR, bpm				
Baseline	78.4 ± 12.4	—	70.6 ± 10.3	—
Hyperoxia	72.8 ± 11.2	<0.001	64.6 ± 7.1	<0.001

*P* values (versus baseline) were obtained with Student's paired *t*-test. —, not applicable.

### Retinal Vascular Responses to Hyperoxia

Table 3 shows the changes in RPC vessel density after hyperoxia in patients with POAG and healthy controls. The perfused vessel density decreased significantly in the whole image, the inside disc, and the peripapillary area after oxygen inhalation in healthy controls (all *P* < 0.001). In patients with POAG, a significant reduction of RPC vessel density was observed after hyperoxia in the whole image (*P* = 0.02) and the peripapillary region (*P* = 0.001) compared to baseline. There was a slight reduction in the inside disc region; however, this difference was not statistically significant (*P* = 0.06).

**Table 3.** Retinal Perfused Vessel Density at Baseline and after Hyperoxia

	POAG, n = 27		Control, n = 14	
Characteristic	Mean ± SD	*P* Value	Mean ± SD	*P* Value
Whole image				
Baseline	41.7 ± 4.9	—	50.8 ± 3.0	—
Hyperoxia	40.9 ± 4.5	0.02	47.8 ± 3.1	<0.001
Inside disc				
Baseline	44.8 ± 7.8	—	49.6 ± 4.9	—
Hyperoxia	43.9 ± 8.6	0.06	46.1 ± 5.7	<0.001
Peripapillary				
Baseline	44.7 ± 6.1	—	54.2 ± 4.1	—
Hyperoxia	43.2 ± 5.4	0.001	51.0 ± 4.4	<0.001

*P* values (versus baseline) were obtained with Wilcoxon's signed rank-sum test. —, not applicable.

### Retinal Vascular Response Changes in Patients with POAG

Table 4 presents alterations in retinal microvascular reactivity in patients with POAG compared with healthy controls. Although a reduction in RPC vessel density in response to hyperoxia was also observed in patients with POAG (Table 3), the differences in RPC vessel density between baseline and the hyperoxia condition were significantly larger in the healthy controls. These differences were apparent in both the absolute and relative differences in the whole image (*P* < 0.001, *P* = 0.004), the inside disc (*P* = 0.006, *P* = 0.02), and the peripapillary region (*P* = 0.006, *P* = 0.04). The OCTA images of RPC in the ONH area at baseline and during the hyperoxia test in the two groups are shown in Figure. These results indicate that the ability of retinal microvessel autoregulation significantly decreased in patients with POAG.

**Table 4.** Changes in Retinal Vascular Response to Hyperoxia in Patients with POAG Compared with Healthy Controls

Characteristic	POAG, n = 27	Control, n = 14	*P* Value
Absolute difference^*^			
Whole image	0.9 ± 1.7	2.9 ± 1.3	<0.001
Inside disc	0.9 ± 2.3	3.6 ± 2.8	0.006
Peripapillary	1.5 ± 2.0	3.2 ± 1.2	0.006
Relative difference, %^†^			
Whole image	1.9 ± 4.4	5.8 ± 2.5	0.004
Inside disc	2.4 ± 5.7	7.3 ± 5.7	0.02
Peripapillary	3.0 ± 4.6	6.0 ± 2.4	0.04

Data are expressed as mean ± standard deviation. *P* values were obtained with Wilcoxon's rank-sum test.

* Absolute difference: value of baseline minus hyperoxia.

† Relative difference: ratio of absolute difference and baseline value (%).

## Discussion

This study used OCTA and oxygen inhalation tests to investigate retinal vasoreactivity of the optic disc microcirculation in patients with POAG. OCTA allows for the acquisition of accurate retinal microvasculature images in a fast, safe, and noninvasive manner. We found that, similar to healthy participants, the retinal microvessels in patients with POAG also responded to hyperoxia provocation. However, this vasoreactivity was impaired compared to healthy controls.

The baseline blood flow characteristics of the included patients with POAG and healthy controls were similar except for PR. The PR of the POAG group was higher than that of the control group, even though the two groups were precisely sex and age matched and the inclusion and exclusion criteria were strictly followed. To confirm that the differences in retinal vascular perfusion and responses to hyperoxia between the two groups resulted from POAG but not the difference in baseline PR, we conducted univariate linear regression to evaluate the effect of basic PR on retinal vessel density and vasoreactivity. We found that PR was not associated with our primary outcomes. Therefore, we believe that the baseline PR difference between the POAG and control groups did not confound the effects of POAG on which we focused.

After the hyperoxia test, the PR of both groups was significantly decreased compared with the baseline, while no significant changes were observed in SAP, DAP, MAP, and MOPP. The systemic responses to hyperoxia in healthy and POAG participants in our study agreed with those detailed in previous reports.^25^^,^^26^ The systemic blood flow responses to hyperoxia in patients with POAG were consistent with the healthy controls, suggesting that the changes of retinal vascular reactivity we observed in patients with POAG were primary to eye disorders but not secondary to possible changes in systemic hemodynamic factors.

Consistent with the previous studies, the perfused vessel density of RPCs in the optic disc area of patients with POAG was significantly lower than in healthy controls.^27^^–^^29^ Several studies have investigated the reduction of retinal perfused vessel density in POAG using OCTA, while to the best of our knowledge, no study has explored the retinal vasoreactivity in patients with glaucoma. This new technology has been applied to study retinal vascular responses in healthy participants^18^^,^^25^^,^^30^ and patients with diabetes.^31^^–^^33^ In this study, we observed a distinct retinal vessel response to hyperoxia in healthy participants, corresponding to previous research.^18^^,^^25^^,^^30^ More important, we further detected an existent but impaired retinal vasoreactivity in patients with POAG. We found that the retinal vascular perfusion was still significantly reduced after the hyperoxia test in patients with POAG, but this change was significantly smaller than that of healthy controls. We expanded the use of OCTA for studying retinal vasoreactivity from healthy people to patients with glaucoma, which we believe can contribute to increasing the use of OCTA in other retinal functional analysis.

Vasoreactivity is thought to be of great importance in POAG cases. However, previous studies have mostly focused on the large intracranial vessels. Using transcranial Doppler, Harris et al.^26^ found that the vasoreactivity to hyperoxia in the middle cerebral artery was absent in patients with POAG, and Hosking et al.^34^ observed similar results in ophthalmic arteries in patients with glaucoma. Consistent with previous studies, we found that the vascular reactivity of retinal RPCs was impaired in POAG. However, the perfused vessel density of RPCs still significantly decreased after oxygen inhalation, indicating that the capillaries around the optic disc could still respond to hyperoxia. This discrepancy may be due to the different testing equipment. Compared to Doppler, OCTA may more sensitively detect differences in blood flow perfusion between baseline and hyperoxia. In addition, the OCTA software we used in this study is more sensitive than previous versions. By contrast, the changes in capillary perfusion under different gas conditions may be more detectable than in noncapillaries. The most important factor we attribute to this inconsistency is the difference in the vasoreactivity impairment of POAG between the large arteries and capillaries. A previous study indicated that capillary responses contributed to more than 80% of cerebral blood flow changes, suggesting that the capillary system has a large capacity for vasoreactiviy.^35^ In patients with POAG, the vasoreactivity of the large vessels may be more vulnerable to impairment than capillaries, which have a relatively high compensation ability. Therefore, although the retinal vasoreactivity of the RPCs around the ONH was significantly decreased in POAG, the RPCs could still respond to hyperoxia by reducing the ONH perfusion. In addition, most patients with POAG enrolled in our study were in the early stages (21 eyes) and midstages (4 eyes) of POAG. Whether the retinal microvascular response would disappear in late-stage patients with POAG is unknown and needs further investigation.

Our study has several limitations. First, because the included patients with POAG were not newly diagnosed, the use of antiglaucoma agents might have affected the retinal vasoreactivity results. However, antiglaucoma drops were used continually once the patient was diagnosed with POAG unless surgical treatment was performed. Therefore, the results of our study may better reflect and correspond to clinical practice. The second limitation of our study is that OCTA measures perfused vessel density, not absolute blood flow. The alteration of capillaries measured by OCTA before and after hyperoxia reflects the changes in the nature of blood flow through the microvessels, not the absolute presence or absence of the microvessels. Nevertheless, OCTA has been extensively applied in glaucoma research and clinical treatment, and we believe our results are convincing and meaningful. Finally, the sample size of our study was relatively small and limited to Chinese adult participants. Due to the small number of patients with POAG, it is hard to make further subdivisions based on glaucoma severity for further analysis. Larger studies with larger cohorts of various ethnic backgrounds and patients of different glaucoma stages will shed more light on the retinal microvascular autoregulation of POAG.

In conclusion, our study used OCTA to explore the retinal vasoreactivity in optic disc capillaries in patients with POAG. The perfused vessel density of the RPCs was decreased in response to hyperoxia, while the changes between hyperoxia and baseline were significantly smaller than in healthy participants. These results demonstrate that the retinal autoregulation of microvasculature in patients with POAG was impaired. Our study highlights the usefulness and feasibility of OCTA for studying retinal vascular autoregulation in glaucoma, which may be conducive to the detection of glaucoma progression and advance our understanding of the pathophysiology of POAG.

## Supplementary Material

Supplement 1
