# Supplementary material for: Retinal Microcirculatory Responses to Hyperoxia in Primary Open-Angle Glaucoma Using Optical Coherence Tomography Angiography
Source: Invest Ophthalmol Vis Sci. 2021 Nov 3;62(14):4. doi: 10.1167/iovs.62.14.4 (PMC8572508; doi:10.1167/iovs.62.14.4)
Supplement: Supplement 1 [file iovs-62-14-4_s001.pdf]

Supplementary Table S1 Univariate linear regression results of associations between pulse rate and OCTA vessel density measurements

**Variables**	β	***P* Value**	R^2
Baseline			
wi-PVD	-0.03	0.71	0.004
id-PVD	0.08	0.38	0.02
peri-PVD	-0.04	0.71	0.004
Absolute Δ*			
wi-PVD	-0.02	0.47	0.01
id-PVD	0.008	0.83	0.001
peri-PVD	-0.02	0.44	0.02
Relative Δ*			
wi-PVD	-0.04	0.47	0.01
id-PVD	0.01	0.90	<0.001
peri-PVD	-0.04	0.48	0.01

PVD, perfused vessel density; wi-PVD, whole image PVD; id-PVD, inside disc PVD; peri-PVD, peripapillary PVD

*Δ: Difference
